# Advances in Screening for Radiation-Associated Cardiotoxicity in Cancer Patients

**DOI:** 10.1007/s11886-023-01971-x

**Published:** 2023-10-05

**Authors:** Walter Schiffer, Lauren N. Pedersen, Matthew Lui, Carmen Bergom, Joshua D. Mitchell

**Affiliations:** 1 Cardiovascular Division, Department of Medicine, Washington University School of Medicine, 660 S. Euclid Ave, CB 8086, St. Louis, MO 63110 USA; 2 Cardio-Oncology Center of Excellence, Washington University School of Medicine, St. Louis, MO USA; 3 Department of Radiation Oncology, Washington University School of Medicine, St. Louis, MO USA; 4 Alvin J. Siteman Cancer Center, Washington University in St. Louis, St. Louis, MO USA

**Keywords:** Radiation, Cardiotoxicity, Cancer, Cardio-oncology, Biomarkers, Screening, Risk prediction

## Abstract

**Purpose of Review:**

Radiation is foundational to the treatment of cancer and improves overall survival. Yet, it is important to recognize the potential cardiovascular effects of radiation therapy and how to best minimize or manage them. Screening—both through imaging and with biomarkers—can potentially identify cardiovascular effects early, allowing for prompt initiation of treatment to mitigate late effects.

**Recent Findings:**

Cardiac echocardiography, magnetic resonance imaging (MRI), computed tomography, and measurements of troponin and natriuretic peptides serve as the initial screening tests of choice for RICD. Novel imaging applications, including positron emission tomography and specific MRI parameters, and biomarker testing, including myeloperoxidase, growth differentiation factor 15, galectin 3, micro-RNA, and metabolomics, hold promise for earlier detection and more specific characterization of RICD.

**Summary:**

Advances in imaging and novel applications of biomarkers have potential to identify subclinical RICD and may reveal opportunities for early intervention. Further research is needed to elucidate optimal imaging screening modalities, biomarkers, and surveillance strategies.

## Introduction

Radiation therapy (RT) remains a key component of treatment for many cancers due to its success in both reducing cancer mortality and recurrence. However, RT has been associated with cardiovascular dysfunction and long-term adverse cardiac events, including ischemic heart disease and cardiac mortality [1]. Though the precise mechanisms of radiation-induced cardiac disease (RICD) are still being elucidated, current evidence suggests that RT directly damages DNA and induces inflammation via the creation of reactive oxygen species. This damage manifests as diffuse interstitial fibrosis of the myocardium and narrowing of arterial and capillary lumens [2]. Injury to capillaries results in microthrombi and occlusion, reduced vascular density, perfusion defects, and ischemia. Subsequently, myocardial injury leads to myocyte death and fibrosis [3]. Downstream effects of radiation therapy include coronary artery disease, with intimal injury leading to the presence of myofibroblasts and platelet deposition and atherosclerosis, valvular dysfunction with fibrosis, diastolic dysfunction due to impaired myocardial compliance mediated by fibrosis, and dysrhythmias due to fibrosis of the native conduction system. Radiation therapy has also been noted to have deleterious pericardial effects, including fibrosis and effusions.

Since the 1990s, when thoracic RT conferred high levels of radiation exposure, approaches to RT have evolved, reducing cardiac exposure by reducing field size and lowering cumulative doses without sacrificing anti-tumor efficacy [4]. Cardiac radiation is typically measured by mean heart dose (MHD), or the average dose received by the whole heart. Risk of RICD increases proportionally with MHD, with a relative risk increase of up to 16% per gray (Gy) MHD [5].

While MHD provides a broad snapshot of the radiation received by the whole heart, RICD appears to have associations based on doses to specific substructures. For example, radiation to the left anterior descending (LAD) has been associated with increased need for intervention for incident mid-LAD stenosis [6]. Similarly, higher doses of left ventricle (LV) radiation have been associated with heart failure and decreased LV ejection fraction [7], in addition to overall mortality [8–11]. Pulmonary artery [12, 13], proximal superior vena cava [13], and left atrium radiation dosing have been found to correlate with RICD and cardiac survival [14–16]. Valvular and coronary artery origin dosing have also been associated with coronary stenosis and valvular heart disease [17, 18]. Lastly, pericarditis is associated with the total radiation dose and delivery [19, 20], usually with higher doses of at least 40 Gy of radiation [21].

As the efficacy of cancer therapy improves, the importance of managing cardiovascular risk factors has become paramount to improving overall survival. In older patients treated for 9 common cancers, cardiovascular mortality was found to confer higher mortality than the primary cancer from the time of cancer diagnosis [22]. The emergence of cardiovascular mortality as a leading cause of death in certain patient cohorts highlights the importance of cardiovascular risk reduction, although this has only occurred due to the progress made in reducing cancer related mortality.

Given the benefit of radiation therapy in treating many cancers, but the increased risk of early and late cardiovascular adverse effects, screening and preventive management of cardiovascular risk is important for long-term patient outcomes. The presence of concomitant risk factors such as combined therapy with anthracyclines, younger age (< 25 years) at time of radiation, previous CVD, diabetes, hypertension, smoking, and pre-existing circulatory or respiratory diseases increase the risk of cardiotoxicity [23]. The International Cardio-Oncology Society (ICOS) expert consensus statement on radiation cardiotoxicity outline general strategies to mitigate cardiotoxicity including screening with annual comprehensive cardiovascular history and physicals, review of available CT images for atherosclerotic calcifications, and optimization of cardiovascular risk factors [24]. In patients who undergo head and neck radiation, further assessment for orthostasis and carotid disease can be done by physical exam, carotid ultrasound at 1 year after radiation in high-risk patients, and carotid ultrasounds every 5 years in all patients. In patients who undergo thoracic radiation, the history and physical should also consider the risk of superior vena cava obstruction or subclavian artery stenosis including bilateral blood pressure measurement. High-risk patients should get a transthoracic echocardiogram (TTE) at 6–12 months after radiation therapy, and all patients with the heart in the radiation field should undergo ongoing screening with TTE and possible ischemic evaluation at approximately 5-year intervals.

Notably, these recommendations do not incorporate different screening intervals for patients with different MHD. The ICOS recommendations mention that patients who undergo significant thoracic radiation (> 30 Gy) with the heart in the treatment field are considered high-risk, but this may not be granular enough to capture the dose–response cardiac risk of radiation.

**Fig. 1:**
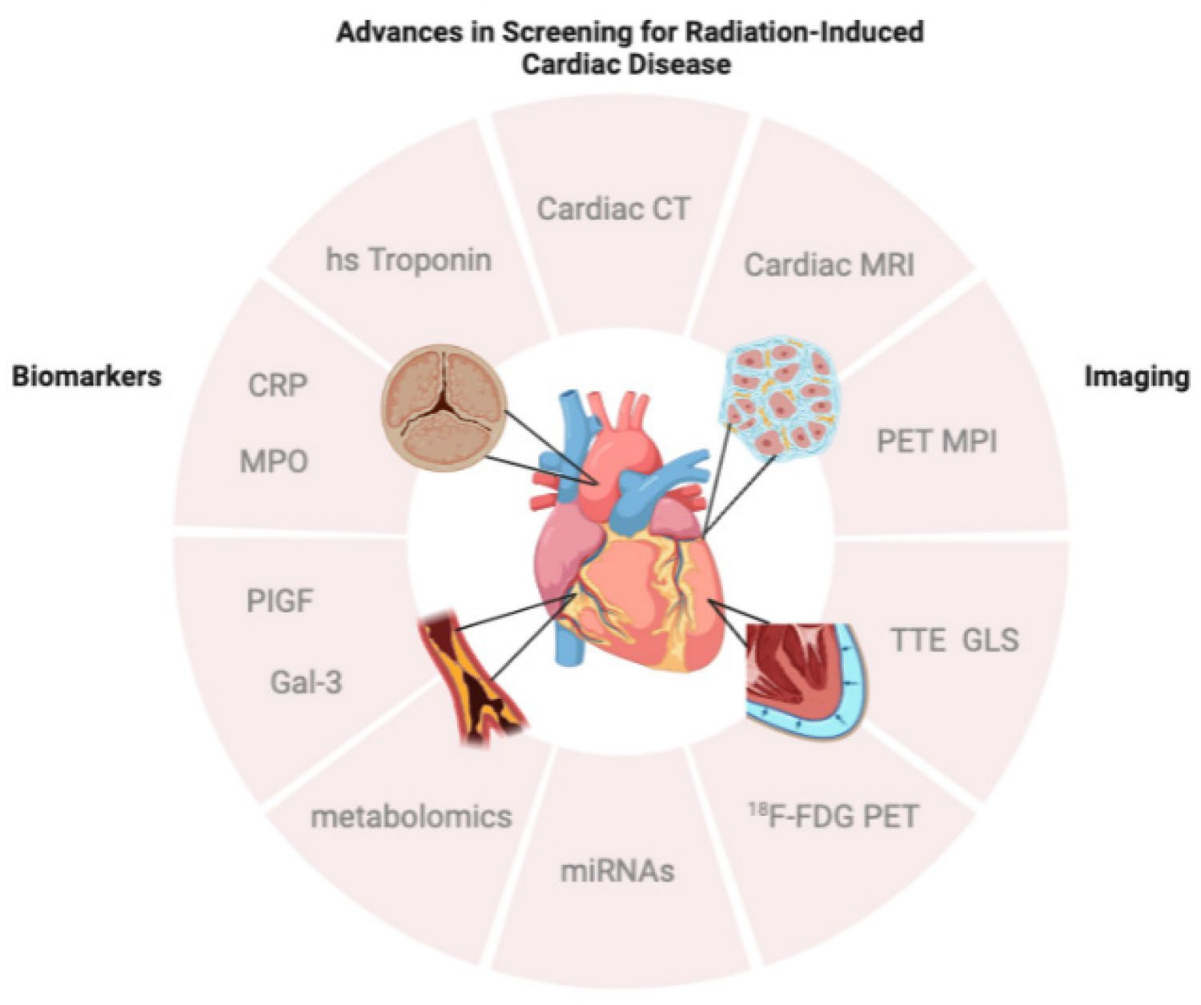
Novel applications of multimodality cardiac imaging and biomarkers drive advances in screening for radiation-induced cardiac disease through earlier and more specific recognition of its multiple manifestations, including cardiac fibrosis, valvular heart disease, coronary artery disease, and pericardial disease. CT, computed tomography; MRI, magnetic resonance imaging; PET, positron emission tomography; MPI, myocardial perfusion imaging; TTE, transthoracic echocardiography; GLS, global longitudinal strain; ^18^F-FDG, fluorine 18 fluorodeoxyglucose; miRNA, micro-ribonucleic acid; PIGF, placental growth factor; Gal-3, galectin-3; MPO, myeloperoxidase; CRP, C-reactive protein; hs, high sensitivity

As new research continues to expand our understanding of RICD with respect to time course, mechanisms, and interplay with other risk factors, new tools are evolving to help capture RICD at earlier stages. Multimodality imaging and biomarker analysis serve as two central avenues in the advancement of screening for RICD and will each be discussed in detail (Fig. 1).

## Advances in Imaging for Screening of Radiation-Related Cardiovascular Disease

Multimodality cardiac imaging represents a rapidly developing avenue for screening and monitoring of RICD. Modalities include non-gated CT chest imaging, electrocardiography (ECG)-gated cardiac CT (CCT), positron emission tomography (PET) myocardial perfusion imaging, F-18 fluorodeoxyglucose (FDG) PET, cardiac magnetic resonance imaging (MRI), and transthoracic echocardiography (TTE) with strain imaging. Each of these modalities confers advantages and disadvantages in screening for RICD, depending on patient factors, availability, and cost (Table 1).

**Table 1:** Strengths and weaknesses of established and novel imaging techniques for screening of RICD

Imaging modality	Important parameters	Strengths	Weaknesses	Expert consensus and guideline screening recommendations
Transthoracic echocardiogram	LV ejection fraction Global longitudinal strain (GLS) Tissue Doppler assessment of RV function and LV diastolic function	Reproducible LV systolic and diastolic function assessment Anatomic assessment of heart and pericardium Widely available	Reduced accuracy in patients with poor acoustic windows Limited tissue characterization Limited pericardial assessment	ICOS, ESC, ASCO, ESMO
Cardiac MRI	T1/T2 mapping Extracellular volume (ECV) Global and circumferential longitudinal strain (GLS, GCS) Gadolinium enhancement	Excellent functional and anatomic assessment Sensitive assessment for myocardial edema, inflammation, and fibrosis Excellent pericardial assessment	Limited expertise Higher cost	ESC, ESMO
Non-contrast CT including radiation planning CT scans	Coronary artery calcifications	Excellent CV risk prediction Identifies subclinical coronary artery disease No additional or low radiation exposure	Not recommended for symptomatic patients Cannot evaluate for obstructive coronary artery disease Non-ECG-gated CT scans have a 9% false-negative rate	ICOS, ESC, SCCT
Cardiac CT angiography	Fractional flow reserve (FFR)	Excellent anatomic assessment, including coronary arteries and pericardium	Limited by body habitus, irregular and fast heart rates	ICOS, ESC, SCCT
^18^F-FDG PET	^18^F-FDG uptake	May detect myocardial inflammation and assess viability	High cost Limited accessibility and expertise Limited anatomic assessment	No
PET myocardial perfusion imaging	Coronary flow reserve (CFR) Myocardial blood flow (MBF)	May detect preclinical microvasculature abnormalities	High cost Limited accessibility and expertise Limited anatomic assessment	No

*LV* left ventricle, *RV* right ventricle, ^18^*F-FDG* fluorine 18 fluorodeoxyglucose, *PET* positron emission tomography, *MRI* magnetic resonance imaging, *ECG* electrocardiogram, *CT* computed tomography, *ICOS* International Cardio-Oncology Society, *ESC* European Society of Cardiology, *ASCO* American Society of Clinical Oncology, *EMSO* European Society of Medical Oncology, *SCCT* Society of Cardiovascular Computed Tomography

Nearly all patients treated with thoracic radiation therapy undergo baseline non-gated CT chest imaging, which should be leveraged to assess for underlying coronary artery calcium (CAC) according to the recent ICOS consensus statement on RICD (ICOS). CAC may be assessed qualitatively or quantitatively to identify patients with subclinical coronary artery disease (CAD) who are at higher risk for RICD. While traditionally, CAC has been evaluated with formal ECG-gated CT scans, CAC seen on non-ECG-gated non-contrast CT scans, including radiation planning scans, correlates with formal gated studies and has significant predictive value [25, 26]. In a meta-analysis of 3 studies with 661 participants, the agreement between non-gated and ECG-gated CT scans was 0.94 (95% CI: 0.89–0.97) [25]. With fewer image slices in most non-ECG studies, however, there can be a notable false-negative rate of 9% as well as an underestimation of high CAC scores in 19%. In patients without available non-gated CT chest imaging, a formal ECG-gated CAC scan can evaluate for calcified plaque with limited radiation dosing (~ 1 mSv).

CAC is a well-established risk factor for cardiovascular (CV) events in non-cancer patients and has recently been validated in the breast cancer population with the BRAGATSTON trial, which demonstrated that higher CAC, as assessed by automated quantification on RT simulation CT, correlated with future hospitalization for CV events [27]. In this study, patients with high CAC (Agatston score > 400) had a 28.2% CV event rate, as compared to 5.2% in patients with no CAC at a median 51 months of follow-up. Importantly, CAC was most strongly associated with future CV events in those patients who also received anthracyclines. Similar findings were described in a smaller study of patients with non-small cell lung cancer who underwent RT (median dose 74 Gy). Of the 109 patients included, 64 had CAC on baseline CT, and only 16 of these had a formal diagnosis of CAD. RT-related CV events increased in frequency with increasing CAC burden, with 42% of patients with a high CAC burden experiencing one CV event over the course of a median 8.8-year follow-up [28•].

ECG-gated cardiac, or coronary, CT angiography (CCTA) provides improved resolution of the coronary arteries and assesses the burden of calcified and noncalcified plaque. Recent advances also allow for the calculation of fractional flow reserve, which identifies hemodynamically significant coronary obstruction [29]. Together, this information provides detailed anatomic and functional information about specific coronaries arteries. This may prove especially helpful in patients undergoing radiation therapy, as the left anterior descending artery is often exposed to the highest radiation dose [30]. Additionally, CCTA provides detailed cross-sectional images of the pericardium, aortic valve, and ascending aorta, which may aid in the screening of RT-related pericarditis and constriction, aortic valvulopathy, and aortic aneurysm, respectively. CCTA is quickly being adopted as a first-line screening tool for coronary artery disease in symptomatic, intermediate risk non-cancer patients [31]. Because of its utility in assessing multiple potential sequelae of thoracic RT, the ICOS and Society of Cardiovascular Computed tomography consensus statements suggest that CCTA could serve as an initial screening modality for RICD [24, 30], though prospective clinical data is needed to validate its role as an initial screening tool. The information gained by the CCTA relative to other techniques must be weighed against potential side effects of contrast and additional radiation exposure. CCTA has been best studied in evaluating symptomatic patients, such as those with chest pain.

PET with myocardial perfusion imaging allows for the assessment of perfusion defects related to epicardial coronary or microvascular damage as sequelae of RT [32]. Screening for microvascular disease with myocardial perfusion imaging holds promise as an early marker for RICD that has not yet caused LV dysfunction or clinically significant epicardial disease. Several radiotracers (^15^O-H_2_O, ^13^N-NH_3_) may be used with PET to determine coronary flow reserve (CFR), which compares stress and resting myocardial blood flow (MBF) to assess coronary microvascular function. A small number of clinical studies have examined CFR in patients treated with RT with inconsistent results. In one study of 20 patients with breast cancer undergoing RT (48 Gy in 24 fractions), CFR was not significantly different in irradiated versus non-irradiated myocardial segments at a median 7-year follow-up [33]. However, this study was only powered to detect a relatively large (40%) change in MBF, which would be unlikely in the era of modern RT techniques [34]. Another study with 18 patients found decreased MBF in half of patients at 2 months post-RT, with perfusion defects predominantly affecting myocardium supplied by the left anterior descending coronary artery (*p* = 0.032), though perfusion abnormalities did not correspond to clinical symptoms [35]. Larger studies are needed to determine the effect of modern RT protocols on myocardial perfusion, and whether identifying perfusion defects can predict clinically significant RICD.

FDG PET allows for assessment of myocardial inflammation and altered myocardial metabolism, making it a potential tool to detect radiation-related myocardial inflammation prior to onset of LV dysfunction. In a small study of 11 patients undergoing RT for breast cancer, a 10% increase in FDG PET uptake was seen in myocardium supplied by the left anterior descending artery (*p* = 0.04) [36•]. In one study using a preclinical animal model of RICD, increased FDG uptake was seen after a single dose of 20-Gy RT to the anterior myocardium. Increased FDG signal in irradiated dogs corresponded to myocardial perfusion defects at 6 months, as well as a significant reduction in LVEF at 12 months [37]. The EUCLID Trial (PET/CT Imaging to Evaluate Cardiac Radiation Damage in Patients with Lung Cancer) is one ongoing study that aims to identify the relationship between RICD and functional imaging changes on FDG PET and expected to be completed in 2026 (ClinicalTrials.gov ID NCT05775939). This and other ongoing investigations will clarify the prognostic value of FDG PET for RICD.

Cardiac MRI, the current gold standard for LV functional assessment, provides multiple functional (e.g., global longitudinal strain [GLS], global circumferential strain [GCS]) and anatomic cardiac parameters, as well as myocardial tissue characterization, all of which may be employed to assess changes related to RT. To date, studies examining changes on cMRI related to RT have been small and with shorter follow-up. Moreover, tissue characterization with T1 mapping and ECV are very sensitive markers of non-specific myocardial changes related to edema and/or fibrosis and may or may not correlate to long-term LV dysfunction.

In one study of 24 patients undergoing RT for esophageal cancer, 14 patients were found to have increased native T1 values and extracellular volume (ECV, a marker of edema and fibrosis) in the septum at 6 months [38]. At 18 months, LV stroke volume index was reduced along with increased late gadolinium enhancement [38]. Increased ECV in the apical and basal segments (6% and 5%, respectively) was also noted on cMRI at 1 month post-left-sided RT in 15 patients with breast cancer (*p* < 0.02). This same study also noted a 7% reduction in LV stroke volume (*p* < 0.02) [39]. Another small study similarly noted higher T1 values after RT at 6-year follow-up in patients who received higher MHD for breast cancer [40]. By contrast, a recent study of 16 patients undergoing chest RT were not found to have significant changes in LVEF, GLS, GCS, ECV, or T1 and T2 mapping at a relatively short 6 months of follow-up, though a decline in LV GLS over time trended towards significance [41].

**Fig. 2:**
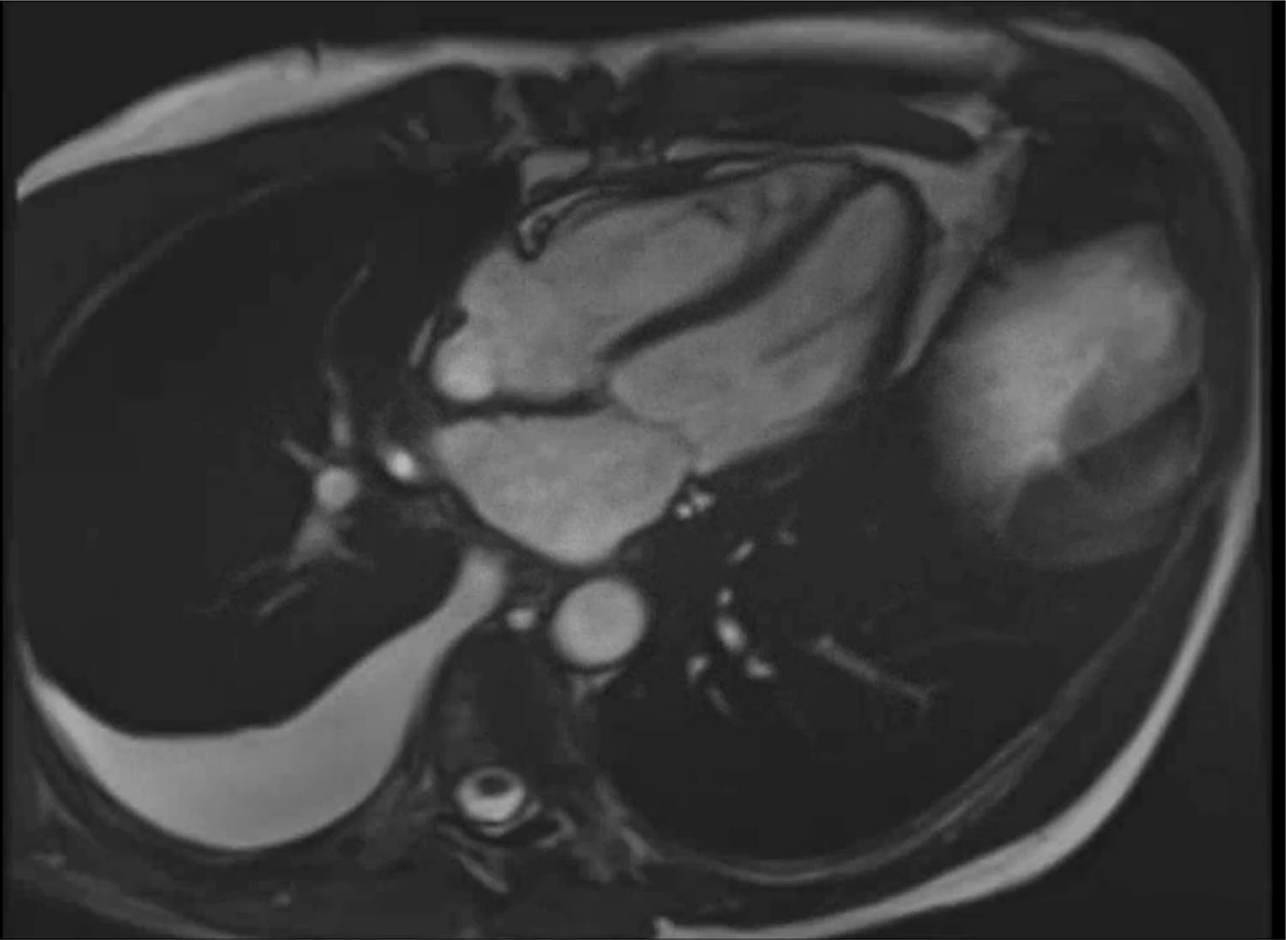
Pericardial constriction in RICD. Cardiac MRI demonstrating pericardial thickening and interventricular dependence in a patient with constrictive pericarditis

Additional studies are needed to examine cMRI changes over extended follow-up to determine which parameters are best able to predict RICD. cMRI is also helpful in detecting pericardial inflammation and fibrosis causing constrictive pericarditis, though the diagnosis of constriction is a clinical one and patients will often be symptomatic. Increased pericardial thickness is often seen on black blood spin echo sequence, and late gadolinium contrast enhancement, increased native T1/T2 mapping values, and high T2-weighted signal intensity can indicate pericardial inflammation (Fig. 2) [42].

TTE serves as the initial screening modality of choice for RICD, and the addition of strain imaging has augmented its sensitivity to detect changes in myocardial function as compared to conventional echocardiographic parameters. The SUCCOUR (Strain Surveillance of Chemotherapy for Improving Cardiovascular Outcomes) prospectively randomized 331 patients receiving cardiotoxic chemotherapy (57% of whom also received thoracic RT) and found that reduced GLS not only predicted systolic LV dysfunction, but also that a GLS-guided approach led to fewer cancer therapy-related CV events [43]. Another study of 40 chemotherapy naïve women treated with RT for breast cancer found a significant decrease (> 10%) in GLS in 20 patients at 12-month follow-up, despite no change in LVEF [44]. By comparison, an analysis of the BACCARAT (BreAst Cancer and CArdiotoxicity Induced by RAdioTherapy) study found that a reduction in GLS > 10% was not significantly associated with MHD or mean LV dose (mean 3.1 and 6.7 Gy, respectively) after adjusting for hormone therapy [45]. It did, however, find a significant association between GLS reduction and patients with at least 20-Gy exposure to LV at 6-month follow-up [45]. Overall, TTE with strain imaging has demonstrated utility in guiding the management of patients receiving cardiotoxic chemotherapy, but more research is needed to establish its role as a first-line screening tool for RICD.

As multimodality imaging continues to evolve and more sensitively detect subclinical RICD, new clinical data is emerging on how to best use these techniques to leverage their unique strengths and weaknesses. In the meantime, major society guidelines continue to help instruct clinicians on the most effective and evidence-based imaging tools to screen for RICD (Table 2).

**Table 2:** Cardio-oncology and oncology society recommendations on imaging and biomarker screening for RICD

Society (reference)	Definition of high-risk patients	Type of screening	Screening interval
European Society of Cardiology (2022)	**Very high risk** > 25 Gy MHD or RT > 15–25 Gy MHD and doxorubicin > 100 mg/m^2^ **High risk** > 15–25 Gy MHD or RT 5–15 Gy MHD and doxorubicin > 100 mg/m^2^	BNP/NT-proBNP	Yearly
		TTE	1, 3, and 5 years after RT, then every 5 years
		Non-invasive CAD screening (CCTA, or functional stress testing with TTE, cMRI, or SPECT)	Every 5–10 years starting 5 years after RT
International Cardio-Oncology Society (2021)	**High risk** > 30 Gy mediastinal RT with heart in treatment field < 30 Gy with anthracycline exposure Higher dose of RT fractions (> 2 Gy/dose) Underlying CV risk factors or CVD	BNP/NT-proBNP	May be considered every 5 years after RT
		CT chest imaging review for CAC	Prior to RT initiation
		TTE	Within 6–12 months after RT if high risk and within 5 years for all others
European Society of Medical Oncology (2020)	**Increased risk** > 10 Gy MHD	BNP/NT-proBNP Troponin	6–12 months and 2 years after RT, periodically thereafter
		TTE or cMRI	May be considered 6–12 months and 2 years after RT, periodically thereafter
American Society of Clinical Oncology (2017)	**Increased risk** > 30 Gy with heart in treatment field < 30 Gy and anthracycline use	TTE	6–12 months after RT

*RT* radiation therapy, *MHD* mean heart dose, *NT-proBNP* N-terminal pro-B-type natriuretic peptide, *TTE* transthoracic echocardiography, *Gy* gray, *CT* computed tomography, *CAC* coronary artery calcification, *cMRI* cardiac magnetic resonance imaging, *SPECT* single-photon emission computerized tomography [77–79]

## Advances in Biomarkers

Fluid biomarker assessment is a promising strategy to enhance detection and risk-stratification of RICD in patients. Analysis of circulating factors within the blood and urine—including proteins, metabolites, and genetic material—offers multiple advantages in the clinical setting, as biofluid sampling is relatively convenient, low-risk, reproducible, and may provide insight as to a patient’s cardiometabolic comorbidities. Like other markers addressed herein, linking fluid biomarkers to radiation-induced cardiac injury is challenging due to patients frequently receiving two or more adjuvant cancer therapies (e.g., chemotherapy, immunotherapy) (Table 3).

**Table 3:** Established and novel biomarkers and their proposed roles in the screening of RICD

Pathway	Biomarker	Proposed role in RICD	Expert consensus and guideline screening recommendations
Myocardial injury	cTnT, hsTnT	Damage to cardiomyocyte	ESMO
	BNP, NT-proBNP	Mechanical stress to myocardium	ICOS, ESC, ESMO
Inflammation	CRP	Acute phase inflammation	No
	MPO	Oxidative stress and inflammation mediated by neutrophils	No
	Galectin-3	Cardiac remodeling and fibrosis	No
	PIGF	Angiogenesis, atherogenesis	No
Omics	Metabolomics	Unique metabolic signatures of cellular oxidative stress and proteolysis	No
	MiRNA	Genetic markers of cardiomyocyte repair, regeneration, and inflammation	No

*cTnT* cardiac troponin T, *hsTnT* high sensitivity cardiac troponin T, *NT-proBNP* N-terminal pro-B-type natriuretic peptide, *CRP* C-reactive protein, *MPO* myeloperoxidase, *PIGF* placental growth factor, *MiRNA* micro-ribonucleic acid, *ICOS* International Cardio-Oncology Society, *ESC* European Society of Cardiology, *EMSO* European Society of Medical Oncology

### Cardiac Troponins and B-type Natriuretic Peptide

To date, cardiac troponins (cTnT) and B-type natriuretic peptide (BNP) are the most widely studied biomarkers of RICD. In non-irradiated populations, troponins and B-type natriuretic peptide are well-validated markers of cardiac damage. Cardiac troponins, particularly cardiac troponin I (cTnI), is released during cardiomyocyte necrosis, while BNP and its precursor molecules (e.g., pro-BNP and N-terminal fragment pro-BNP) are released by cardiomyocytes in responses to increased cardiac mechanical stress. As such, both are considered diagnostic and prognostic factors in patients with ventricular dysfunction and heart failure and are used clinically to guide cardiovascular intervention [46]. Troponin is often more a signal of acute myocardial injury and risk for future cardiac dysfunction and heart failure, while natriuretic peptides are helpful screening tools to help detect early signs of heart failure.

Mixed evidence exists as to the utility of cTnT and BNP in detecting RICD. In patients with lymphoma or thoracic cancers receiving ≥ 30 Gy to 5% of cardiac volume or mean heart dose (MHD) of ≥ 4 Gy (*n* = 19, ~ 90% received chemotherapy), Donovan et al. found no association of high-sensitivity (hs) TnI and hsTnT with cardiac dose, and in breast cancer patients treated with hypofractionated radiation (*n* = 44, ~ 57% received chemotherapy) [47], De Sanctis et al. reported no significant increases in either cTnI or NT-proBNP [48]. Although Gomez et al. found that 2/25 patients receiving 45 Gy to the whole thorax or 20 Gy to the heart had elevated cTnI and BNP at the end of radiation treatment, these changes did not reach statistical significance [49]. Furthermore, while median BNP was significantly elevated in patients at the time of first follow-up (1–2 months post-radiation), this was largely due to BNP levels measured in patients receiving both radiation and chemotherapy [49]. In contrast to the above findings, Palumbo et al. reported significant increases in BNP at 1 and 6 months post-treatment in patients with left-sided breast cancer (*n* = 43) who had not received adjuvant chemotherapy, and all BNP measurements normalized to baseline values were significantly correlated with V20, V2, V30, V45, mean dose, and MHD [50]. Similarly, in 236 survivors of breast cancer who received 4-field 50-Gy radiation (~ 88% also received chemotherapy), high pro-BNP correlated with coronary artery calcium score, a marker of coronary disease [51]. Finally, in a meta-analysis of 4 studies and 172 patients with breast cancer who received radiation therapy only, pooled standard mean difference for BNP suggested increased plasma BNP, particularly in patients with affected left side [52•]. Taken together, these studies point to the potential utility of cTnT and BNP as biomarkers of cardiac injury in patients receiving radiation, perhaps most strongly so in those that have also received adjuvant cancer therapies.

### C-Reactive Protein

Beyond cTnT and BNP, several novel plasma biomarkers are under investigation for the detection and risk-stratification of RICD, including acute phase proteins and inflammatory cytokines. C-reactive protein (CRP) is an acute phase inflammatory protein that has well-validated utility in predicting acute cardiac events (e.g., myocardial infarction, stroke, sudden cardiac death) in non-irradiated populations but is less explored in patients receiving cancer therapies in general and radiation therapy for thoracic cancers in particular [53]. In patients with thoracic cancer who received radiation (*n* = 30, 23% induction chemotherapy, 80% concurrent chemotherapy), Kuo et al. reported only weak associations between CRP and cardiac dosimetry [54]. Similarly, Tjessem et al. found that CRP was increased in the plasma of breast cancer survivors treated with radiation (*n* = 236, ~ 80% chemotherapy), but that CRP did not associate with Agatston score, a measure of coronary artery calcification [51]. Given these findings, elevations in plasma CRP may result from radiation therapy, yet it is unclear how this directly associates with RICD.

#### MPO

Myeloperoxidase (MPO) is a heme-containing peroxidase primarily secreted by neutrophils that is linked to oxidative stress and inflammation [55]. Elevated MPO is linked to a number of disease states, including non-radiation-induced cardiovascular diseases such as coronary artery disease, acute coronary syndrome, and heart failure [55–57]. As such, MPO is an attractive fluid biomarker for the detection of RICD. Multiple studies suggest that increased MPO is associated with systemic cancer therapy-related cardiac dysfunction [58]. For example, in a multicenter study of patients with breast cancer receiving doxorubicin and trastuzumab (*n* = 78), Ky et al. found increased MPO to be robustly associated with cardiotoxicity at 3 months post-treatment [59]. Additionally, in a recent systematic review and meta-analysis conducted by Wu et al. including 8 studies and 1979 patients, elevated MPO following chemotherapy treatment significantly increased risk of cardiotoxicity (HR = 1.16) [60]. To date, strong links between MPO and radiation injury to the heart in patients with thoracic cancers have not been established but the clinical utility of MPO is of continued investigative interest. The clinical utility of MPO is unfortunately limited since the serum must be immediately placed on ice for accurate testing.

### Galectin-3

Tissue remodeling is necessary to repair damaged myocardium; however, excessive collagen deposition with the cardiac tissue can result in fibrosis and subsequent cardiac dysfunction. Galectin-3, a macrophage-secreted beta-galactoside-binding lectin, participates in cardiac fibrosis and has shown great utility as a prognostic indicator in chronic heart failure and cardiometabolic diseases such as diabetes mellitus [59, 61, 62]. Given that fibrosis is a well-established pathophysiological consequence of many cancer therapies and promising preclinical studies, there was initially enthusiasm for galectin-3 as a biomarker of cancer therapy-induced cardiotoxicity. However, studies investigating plasma galectin-3 levels in patients receiving systemic cancer therapies (e.g., anthracycline, doxorubicin, trastuzumab) show disappointing results. In 192 patients with breast cancer treated with anthracycline, de Barros Wanderley et al. found no interaction of plasma galectin-3 with subsequent cardiovascular disease [62]. Similarly, in 78 patients with breast cancer undergoing doxorubicin and trastuzumab, Ky et al. reported that galectin-3 was not altered at 3 months post-treatment [59]. Given these data, more work is needed, specifically in the context of RICD, to establish galectin-3 as a viable biomarker of cancer therapy-induced cardiac dysfunction.

#### PIGF

Placental growth factor (PIGF), a member of the vascular endothelial growth factor (VEGF) family, is an angiogenic and atherogenic growth factor that is linked to ischemic heart disease, heart failure, and, notably, altered cardiovascular dysfunction during pregnancy [63]. Two recent studies suggest that systemic cancer therapies and, more topically, thoracic radiation may increase plasma PIGF, but the relationship between treatment-induced PIGF and cardiac dysfunction is tenuous [59]. In patients with breast cancer receiving chemotherapy (e.g., doxorubicin and trastuzumab), Ky et al. found PIGF to be increased in the plasma of patients at 3 months following treatment; however, plasma PIGF was not associated with cardiotoxicity [59]. In a prospective longitudinal study of patients treated with photon or proton radiation therapy (*n* = 87), Demissei et al. reported plasma PIGF to be increased in patients with lymphoma or lung cancer, but not those in with breast cancer, at a median of 20 days following treatment. In lymphoma and lung cancer patients, follow-up PIGF was independently associated with MHD, V5, and V30 but were not associated with echocardiographic parameters of cardiac function [58]. Given the short duration of follow-up in both of the above studies, more studies with longer follow-up periods may help to establish the potential utility of PIGF as a biomarker of RICD.

### Other Circulating Cytokines, Chemokines, and Growth Factors

Pro-inflammatory cytokines within circulation have also been investigated for their potential utility as biomarkers of cardiac dysfunction following cancer treatments. A number of studies in patients with lung cancer treated with radiation suggest that elevated IL-1B, IL-6, IL-8, TNF-a, and/or TGF-B1 may associate with radiation-induced pneumonitis and/or fibrosis [64–67]; however, less evidence is available concerning injury to the heart. In a small sample (*n* = 17) of patients with lung cancer treated with radiation, Tao et al. found that plasma CCL2, VEGF, IL-1B, and IL-6 tended to be increased after treatment but were not statistically significant [68]. Additionally, changes in VEGF and IL-6 correlated with mean heart dose [68]. Conversely, in patients with thoracic cancers who received radiation (*n* = 30, 23% induction chemotherapy, 80% concurrent chemotherapy), Kuo et al. reported no significant associations of 16 cytokines and chemokines with cardiac dosimetry and in patients with breast cancer previously treated with radiation (*n* = 55) neither TGF-B1 nor IL-6 significantly associated with future risk for RICD [54].

### Metabolomics

Radiation impacts cellular metabolism through both direct and indirect means, and emerging evidence suggests that assessing radiation-induced metabolic changes—so-called metabolomics—may be a useful tool for characterizing RICD. To date, much of this data has been collected in pre-clinical models and a number of metabolites—ranging from products of beta oxidation and ATP synthesis to oxidative stress and proteolysis, have been implicated in the radiation response—as has been detailed in-depth by several recent reviews [69, 70]. Interestingly, Unger et al. recently compared the metabolomic and lipidomic profiles of rats receiving localized cardiac radiation to that of patients with esophageal cancer who underwent radiation therapy [71]. The authors reported that steroid hormone biosynthesis and vitamin E metabolism pathways were found to be altered by radiation in both rats and patients [71]. Given such findings, more translational and clinical research is needed to advance the potential of metabolomics as a biomarker of RICD.

### MicroRNAs

MicroRNAs (miRNAs) are highly conserved, non-coding RNA fragments composed of 19–25 nucleotides that negatively regulate ~ 30% of human gene expression by binding to messenger RNA [72, 73]. A number of miRNAs have been linked to non-cancer treatment-induced cardiac dysfunction, including cardiac hypertrophy (e.g., miR-1, miR-133a), fibrosis (e.g., miR-21), coronary artery disease (e.g., miR-624, miR-340), and chronic heart failure (miR-221, miR-21, miR-409-5p, miR-376a, miR-154) [74]. To date, studies investigating miRNA following systemic therapies in patients with thoracic cancers are more abundant than those exploring radiation-induced miRNA alterations. The breadth of miRNAs implicated in chemotherapy-induced cardiotoxicity is well illustrated by a recent systematic review conducted by Brown et al. In reviewing 98 studies of patients with breast cancer treated with anthracyclines, Brown et al. concluded that 14 of 33 investigated miRNAs could be considered potentially informative of patients’ treatment-induced cardiac dysfunction [75]. The authors reported that miR-29a-3p, miR-199a-3p, miR-1273 g-3p, miR-4638-3p, miR-34a-5p, miR-1, miR-17-5p, miR-19a, miR-122-5p, miR-130a, miR-378, miR-423, miR-499, and miR-885-5p levels were significantly altered by anthracycline treatment in the studies they reviewed, and these miRNAs were proposed to associate with cardiac repair, cardiomyocyte regeneration, inflammatory signaling, cardiac hypertrophy, angiogenesis, coronary disease, cardiomyopathy, heart failure, and acute myocardial infarction [75]. Of note, Hawkins et al. recently evaluated circulating miRNAs in patients with non-small cell lung cancer (*n* = 63) receiving radiation therapy [76]. The authors reported that a 14 miRNA “signature” (miR-100-5p, miR-106b-5p, miR-145-5p, miR-146a-5p, miR-192-5p, miR-195-5p, miR-223-3p, miR25-3p, miR-34a-5p, miR-574-3p, miR885-5p, let-7c, miR-200b-3p, miR-134) was prognostic for a patient’s risk of developing grade 3 or greater RICD, performing as well as prognostic models based on mean heart dose and pre-existing cardiac disease [76]. Such clinical findings suggest that miRNAs hold great promise for risk stratifying patients treated with thoracic radiation, but more work is needed to determine which miRNAs or combination of miRNAs provide most utility.

## Conclusion

Advances in multimodality imaging and novel biomarkers hold promise for earlier and more accurate detection of both subclinical and symptomatic RICD. TTE with strain imaging, PET myocardial perfusion, FDG PET, and cardiac MRI all confer unique advantages to detect myocardial dysfunction and injury more sensitively, though the clinical benefit of this increased screening sensitivity is still under active investigation. Similarly, novel biomarkers, including PIGF, MPO, galectin-3, as well as metabolic and micro-RNA signatures seek to more promptly and precisely recognize the pathophysiologic processes underlying RICD. As these new tools gain traction with the help of clinical trials, the growing armamentarium of screening tools will allow for a more personalized risk assessment for RICD.
